# The emerging insights into catalytic or non-catalytic roles of TET proteins in tumors and neural development

**DOI:** 10.18632/oncotarget.11412

**Published:** 2016-08-19

**Authors:** Hao Lian, Wen-Bin Li, Wei-Lin Jin

**Affiliations:** ^1^ Department of Oncology, Beijing Shijitan Hospital, Capital Medical University, Beijing, P.R. China; ^2^ Institute of Nano Biomedicine and Engineering, Department of Instrument Science and Engineering, Key Laboratory for Thin Film and Microfabrication Technology of Ministry of Education, School of Electronic Information and Electronic Engineering, Shanghai Jiao Tong University, Shanghai, P. R. China; ^3^ National Centers for Translational Medicine, Shanghai Jiao Tong University, Shanghai, P. R. China

**Keywords:** TET proteins, epigenetics, cancer, neural development, glioma

## Abstract

The Ten-eleven translocation (TET) proteins have been recently identified as critical regulators in epigenetic modification, especially in the methylation of cytosine in DNA. TET-mediated DNA oxidation plays prominent roles in a wide variety of physiological and pathological processes, especially in tumor and neural development. TET proteins execute stepwise enzymatic conversion of 5-methylcytosine (5mC) to 5-hydroxymethylcytosine (5hmC), 5-formylcytosine (5fC) and 5-carboxylcytosine (5caC). In addition to the more proverbial enzymatic role of TET proteins, TET proteins also possess non-enzymatic activity, through interacting with some epigenetic modifiers. In this review article, we focus on TET proteins dual activities (catalytic or non-catalytic) in tumor and neural development. Hence, the clarification of TET proteins dual activities will contribute to our further understanding of neural development and may open the possibility of new therapeutic avenues to human tumors.

## INTRODUCTION

**Figure 1:**
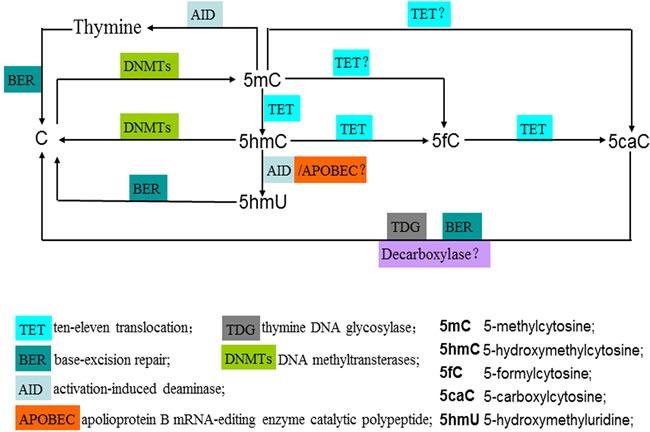
Enzymatic activity of TET family proteins: achieving the dynamic conversion cycle of C, 5mC and 5hmC 5mC is catalyzed by DNA methyltransterases (DNMTs) via the addition of a methylgroup at the 5-position of cytosine. TET family proteins (TET1/2/3) catalyze the stepwise oxidation of 5mC to yield 5hmC, 5fC and 5caC. 5fC and 5caC are able to be recognized and excised by thymine DNA glycosylase (TDG) and replaced with unmethylated cytosines through the base excision repair (BER) machinery, leading to the active demethylation. An alternative active demethylation pathway may be achieved via deamination of 5hmC to 5-hydroxymethyluridine (5hmU) by AID/APOBEC enzymes, followed by BER. However, this deamination mechanism is controversial. During DNA replication, the downregulation or loss of the maintenance activity of DNMT1 dilutes the level of 5mC after each cell cycle, which results in passive demethylation. DNMT3A and DNMT3B could display a reduction-oxidation state-dependent DNA dehydroxymethylase activity, opening a simple pathway for the direct conversion of 5hmC to C. The stem-cell nuclear extracts could be capable of achieving the decarboxylation of 5caC, indicating the stem-cell nuclear extracts may be a decarboxylase. In a proportion of gliomas and breast cancers, a possible mechanism that leads to decreased levels of 5hmC by Eleftheriou et al. is that TET proteins are preactivated in certain cancer-related environment resulting in the preferential catalytic conversion of 5mC to 5caC and 5fC, instead of 5hmC.

Epigenetic mechanisms, mainly including DNA methylation such as covalent modification of cytosine bases leading to 5-methylcytosine (5mC) and histone modifications, are fundamental to regulate many biological and pathological processes in gene expression, cellular differentiation, embryogenesis, neural development and carcinogenesis. TET1 protein was initially described as a fusion partner of the mixed lineage leukemia (MLL) gene in acute myeloid leukemia (AML) as well as pediatric leukemias bearing the t(10:11)(q22;q23) translocation [1–3]. A recent study discovered that human TET1 protein could catalyze the conversion of 5mC of DNA to 5-hydroxymethylcytosine (5hmC), indicating that DNA demethylation may be a TET1-mediated process [4]. Its family members, TET2 and TET3 also have the capacity to catalyze a similar reaction [4–8]. Currently, an accumulating body of evidence mainly involves the enzymatic functions of TET proteins. The catalytic mechanism of TET family proteins is that they not only catalyze the conversion of 5mC to 5hmC, but also further catalyze the conversion of 5hmC to 5-formylcytosine (5fC) and 5-carboxylcytosine (5caC), which are subsequently recognized and cleaved by thymine DNA glycosylase (TDG) and the base-excision repair (BER) process in an active DNA demethylation manner [4–8] (Figure 1). Alternatively, 5hmC may be further deaminated to produce 5-hydroxymethyluridine (5hmU) by activation-induced deaminase/apolioprotein B mRNA-editing enzyme catalytic polypeptide (AID/APOBEC) deaminases [9, 10] (Figure 1). Extensive studies were focused on the catalytic enzymatic roles of TET family proteins; while some investigations illustrated a non-catalytic activity of TET proteins. Several studies have reported that TET proteins interact with other epigenetic modifiers and some transcriptional regulators, such as the histone deacetylase 2 (Hdac 2), the O-glcNAC transferase (OGT), the Sin 3A complex and hypoxiainducible factors (HIFs), independent of their enzymatic activity [12–16]. Furthermore, Cartron et al. observed that TET1 interacts with MeCP2, HDAC1/6/7, EZH2, mSin3A, PCNA, and LSD1 to control its DNA-demethylating function [11]. Thus, TET family proteins act as transcriptional activation or repression of target genes through their enzymatic and non-enzymatic activity in lots of cellular processes. This review outlines recent advances with respect to the catalytic and non-catalytic roles of TET proteins in human tumors and neural development. The elucidation of dual roles of TET proteins will facilitate the better understanding of the process of neural development, tumorigenesis and tumor progression.

## 5HMC LEVELS IN TUMORS AND NEURAL DEVELOPMENT

It is well known that TET proteins catalyze the oxidation of 5mC to generate 5hmC and thereby 5hmC levels reflect diverse functions of TET proteins. Some studies have revealed that 5hmC levels are reduced in hematological malignancies [17, 18]. A broad spectrum of solid tumors, including colon, breast, liver and prostate tumors, reportedly has low levels of 5hmC compared with normal tissues [19–22] (Table 1). For example, Kraus et al. reported that (i) 5hmC concentrations in brain tumors were distinctly decreased compared with those in normal brain tissues; (ii) levels of 5hmC correlated with WHO grade of brain tumors; (iii) 5hmC levels were associated with isocitrate dehydrogenase 1 (IDH1) mutations in diffuse and anaplastic astrocytomas but not in glioblastoma multiforme (GBM) [23]. However, one study's findings showed that 5hmC distributions were significantly higher in diffuse intrinsic pontine glioma (DIPG) compared to adult GBM and pediatric GBM [24]. Furthermore, Takai et al. demonstrated that proneural glioblastomas harbor elevated levels of 5hmC and TET1 [25].

**Table 1:** The enzymatic activity of TET family proteins in neural development

TET proteins	Role in neural development	Intervention method	Ref
TET1	OL development	siRNA	[67]
	neurogenesis	shRNA	[27]
	adult hippocampal neurogenesis and cognition	KO	[65]
TET2	OL development	siRNA	[67]
	neurogenesis	shRNA	[27]
TET3	mOSN development	overexpression	[64]
	OL development	siRNA	[67]
	neurogenesis	shRNA	[27]
	Xenopus eye and neural development	cKO	[66]

Note: OL: Oligodendrocyte; mOSN: mature olfactory sensory neuron; shRNA: short hairpin RNA; siRNA: small interfering RNA; KO: Knockout; cKO: conditional knockout

5hmC has been shown to be abundant in brain tissue: in DNA from human brain cortex, 5hmC levels are about 1% of all cytosines, indicating that 5hmC plays an important functional role in the mammalian brain [26]. Hahn and colleagues have shown that 5hmC levels increase during neuronal differentiation [27]. In other words, the level of 5hmC is enriched in terminally differentiated neurons, whereas less differentiated neurons, neural precursor cells (NPCs) and neural stem cells (NSCs) have lower 5hmC levels.

Taken together, 5hmC levels differ according to different tumor types, tumor grades and neural developmental cell stages. Even in different molecular subtypes of the same tumor, there are contradictory reports on 5hmC levels. All of these findings suggest that TET family proteins play different roles based on distinct human tumors and stages throughout neural development. In this review, we highlight the dual roles of TET family proteins in human tumors and neural development: enzymatic function and non-enzymatic function.

## STRUCTURE OF TET FAMILY PROTEINS

Three members of the mammalian TET gene family have been defined: TET1, TET2 and TET3 [4, 28]. The TET gene underwent triplication and then generated TET1, TET2 and TET3 in jawed vertebrates [29]. All TET proteins contain a similar catalytic C-terminal CD domain, which is composed of a double-stranded β-helix (DSBH) fold and a cysteine-rich (Cys) region [30, 31]. Hu et al. reported the crystal structure of human TET2-DNA complex [32]. This structure suggests that two zinc fingers bring the DSBH and Cys-rich domains together to facilitate a compact catalytic domain formation. These CD domains of TET proteins confer α-ketoglutarate (α-KG)-and iron (II)-dependent dioxygenase activity [4]. TET family proteins convert 5mC to 5hmC through these CD domains, andα-KG is regarded as a co-substrate for enzymatic activity. Zhang and colleagues reported that TET proteins also oxidize 5mC and 5hmC into 5fC and/or 5caC by these CD domains, but the levels of 5fC and/or 5caC are very low [28]. One recent study showed that TET2 interact with Hdac1/2 *via* its DSBH domain [12]. In addition, the catalytic DSBH domain of TET2 is also able to interact with O-linked N-acetylglucosamine (O-GlcNAc) transferase (OGT) [13]. These studies suggest that TET proteins may interact with other epigenetic modifiers through the DSBH domain.

Moreover, Xu and colleagues demonstrated that these CD domains of TET family proteins substantially convert 5mC and 5hmC to 5caC in the presence of ATP *in vitro* [7]. In addition, TET proteins possess the CXXC zinc finger domain at the amino-terminal region; the CXXC domain can be found in TET1 and TET3, but not TET2 [33, 34]. Several studies reported that the CXXC domain of TET1 recognize unmodified cytosine, 5mC and 5hmC, and it prefers to bind to regions in the genome of high CpG content [35, 36]. During evolution, the TET2 gene splitted into two segments encoding catalytic domain and CXXC domain, respectively. The catalytic domain evolved into TET2, whereas the CXXC domain evolved into a distinct gene, IDAX (also known as CXXC4). IDAX could lead to downregulation of TET2 protein *via* activation of caspase 3 [37]. TET2 binds different sets of genomic regions, which is depending on whether IDAX is present [29]. IDAX has previously been indicated as an inhibitor of the Wnt signaling pathway that play an important role in modulating cell proliferation, invasion and survival [38, 39]. Unlike TET2 and TET3, TET1 contains three candidate bipartite nuclear localization signal at its amino-terminus [2], indicating a mainly nuclear location of the protein.

## ENZYMATIC ACTIVITY OF TET FAMILY PROTEINS: ACHIEVING THE DYNAMIC CONVERSION CYCLE OF C, 5MC AND 5HMC

All three TET proteins possess an intrinsic enzymatic capability to catalyze the successive oxidation of 5mC to yield 5hmC, 5fC and 5caC (Figure 1). The TET proteins enzymatic function has opened up new ways for achieving the dynamic conversion cycle of C, 5mC and 5hmC and regulating the balance of DNA methylation and DNA demethylation (Figure 1). Over the past several decades, DNA methylation at the 5′carbon of the cytosine base (5-methylcytosine, 5mC) has been identified as an important epigenetic modification that plays a vital role in some cellular and molecular mechanisms such as establishment of cellular identity, control of gene expression, suppression of transposon elements, genomic imprinting, X-chromosome inactivation and carcinogenesis [8, 40]. DNA methylation is catalyzed by DNA methyltransterases (DNMTs) through the addition of a methylgroup at the 5-position of cytosine, resulting in 5-methylcycosine (5mC). DNMT3A, DNMT3D and DNMT3L create DNA methylation, while DNMT1 maintain the patterns of methylation [41–44]. During DNA replication, the downregulation or loss of the maintenance activity of DNMT1 dilutes the level of 5mC after each cell cycle, which results in passive demethylation. For instance, 5hmC represses binding of the DNMT1/UHRF1 complex to DNA for methylation maintenance [45, 46]. Chen and co-workers reported that the vertebrate denovo DNA methyltransferases DNMT3A and DNMT3B could also exhibit a reduction-oxidation state-dependent DNA dehydroxymethylase activity, opening a simple pathway for the direct conversion of 5hmC to C [47] (Figure 1).

Many studies showed that there might be multiple pathways or mechanisms by which TET family proteins regulate the cycle of DNA demethylation. The TET oxidation products 5-formylcytosine (5fC) and 5-carboxylcytosine (5caC) are able to be recognized and excised by thymine DNA glycosylase (TDG) and replaced with unmethylated cytosines through the base excision repair (BER) machinery, leading to the active demehylation [5–7] (Figure 1). In addition, an alternative active demethylation pathway may be achieved *via* deamination of 5hmC to 5-hydroxymethyluridine (5hmU) by activation-induced deaminase/apolioprotein B mRNA-editing enzyme catalytic polypeptide (AID/APOBEC) enzymes, followed by BER [9, 10] (Figure 1). However, this deamination mechanism is controversial because of no direct biochemical evidence indicates that these deaminases have their robust activity against 5hmC [10]. Furthermore, Schiesser et al. found that the stem-cell nuclear extracts could be capable of achieving the decarboxylation of 5caC, indicating the stem-cell nuclear extracts may be a decarboxylase [48] (Figure 1).

## THE ENZYMATIC ACTIVITY OF TET FAMILY PROTEINS IN TUMORS

**Figure 2:**
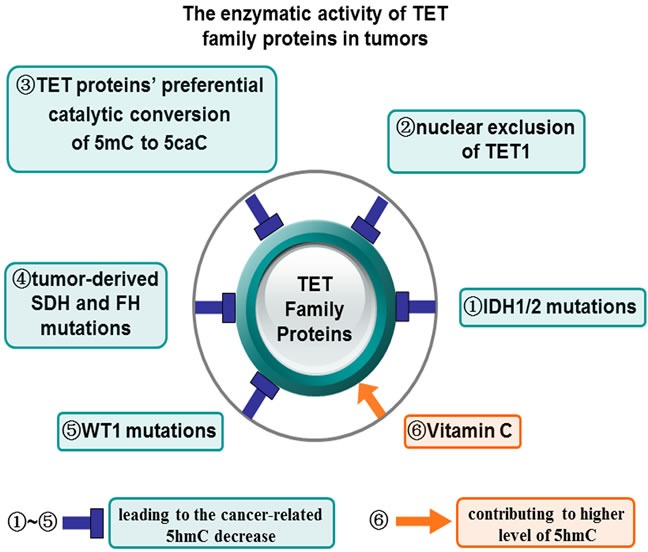
The enzymatic activity of TET family proteins in tumors There are several different mechanisms were involved in the regulation of the catalytic enzymatic activity of TET proteins in tumors. For example, both the tumor-associated IDH1/2 mutations (1) and nuclear exclusion of TET1 inhibit TET1 catalytic activity and decrease 5hmC levels (2). TET proteins exert the preferential catalytic conversion of 5mC to 5caC, instead of 5hmC (3). Tumor-derived SDH and FH mutations also repress TET proteins enzymatic activity and reduce 5hmC levels (4). WT1 mutations indirectly repress TET2 enzymatic activity and reduce 5hmC levels (5), while pretreatment of Vitamin C increase the enzymatic activity of TET family proteins and 5hmC levels (6). TET proteins enzymatic activity and their oxidation product 5hmC can be reduced (1, 2, 3, 4, 5, 6) and increased (6) in the presence of distinct regulatory elements.

To date, several different mechanisms are reported to be involved in the regulation of the enzymatic activity of TET proteins, which are summarized in Figure 2. The point mutants of isocitrate dehydrogenase type 1 (IDH1) and type 2 (IDH2) were frequently found in human tumors, which exhibits lack of normal enzymatic activity, responsible for 2-oxoglutarate (α-ketoglutarate; 2OG) production [49]. The tumor-associated IDH1/2 mutations gain the ability to generate (R)-2-hydroxyglutarate ((R)-2-HG). 2HG represses the TET-enzymatic reaction *via* reduction of the key cofactor 2OG and direct impairment of TET catalytic activity by 2HG [50, 51]. For instance, Xu et al. showed that reduced 5hmC levels in a mixture of Grade III astrocytomas and GBMs containing mutant IDH1 [52]. Since it possesses three nuclear localization signals [2], TET1 may be a mainly nuclear localization enzyme and its enzymatic activity of the conversion of 5mC to 5hmC may be a nuclear event. Indeed, in human embryonic kidney cells and human colon cancer cells, overexpressed TET1 and TET2 are predominantly localized in the nucleus and nuclear TET proteins are gradually translocated to the cytoplasm when they are co-expressed with Aid [53]. In colorectal cancer cells, nuclear expression of TET2 is absent and nuclear export inhibitor can increase the 5hmC level [54]. Müller et al. reported that nuclear exclusion of TET1 is related to reduction of 5hmC in IDH1 wild-type gliomas [55]. These findings showed nuclear exclusion of TET proteins may provide a novel mechanism for the reduction in 5hmC from the tumor cells, which is independent from IDH1 status. Moreover, Eleftheriou and co-workers demonstrated that 5caC levels are elevated in a proportion of gliomas and breast cancers. A possible mechanism that leads to decreased levels of 5hmC proposed by Eleftheriou is that TET proteins are preactivated in certain cancer-related environment resulting in the preferential catalytic conversion of 5mC to 5caC, instead of 5hmC [56]. Recently, Xiao et al. showed that tumor-derived SDH and FH mutations also inhibit TET proteins enzymatic activity *via* accumulation of their respective substrates, succinate and fumarate [57].

Vitamin C is a vital antioxidant that turns Fe(III) to Fe(II) and thereby facilitates the enzymatic integrity of Fe(II)-dependent TET enzymes. Vitamin C has been reported to increase the enzymatic activity of TET family proteins *in vitro* and vivo [58–60], possibly through binding to the catalytic domain of TET enzymes and directly enhancing their enzymatic activity [58]. Treatment with vitamin C was showed to contribute to higher level of 5hmC in melanoma cell lines and to decrease the degree of malignancy by potentiating TET enzymatic activity [61]. Interestingly, Rampal et al. have found that WT1 mutations reduce TET2 enzymatic activity and then result in decreased 5hmC levels in AML. This study has shown that WT1 interact with TET2 and TET3, and may stimulate TET proteins enzymatic activity at specific sites in the genome [62].

These lines of evidences support the notion that multiple pathways inhibit the TET proteins enzymatic activity in various malignancies and thus lead to the cancer-related 5hmC decrease suggesting that reduced 5hmC may be a valuable tumor hallmark for the early diagnosis and prognosis of cancers. Therefore, the treatment that boost the TET proteins enzymatic activity, such as using vitamin C and promoting WT1 overexpression, is likely to be beneficial to tumor treatment.

## THE ENZYMATIC ACTIVITY OF TET FAMILY PROTEINS IN NEURAL DEVELOPMENT

In 2009, a seminal paper reported the high levels of 5hmC occur in neurons [63], pointing to a potential role for TET enzymes in the control of neural development. Hahn and colleagues described the roles of TET proteins and 5hmC during neurogenesis [27]. They have reported that the levels of 5hmC elevate during neuronal differentiation and enrichment of 5hmC is not at enhancers but preferentially at gene bodies of many critical neuronal differentiation-related genes. Transcriptional analysis revealed that accumulation of intragenic 5hmC accompanied with loss of H3K27me3 is involved with gene activation during neuronal differentiation. Polycomb complex modulates the switch of NPCs from expansion to differentiation, while TET enzymes are correlated with maintaining the proper progression of the neuronal differentiation process through TET- mediated formation of 5hmC along gene bodies after this process initiation. In addition, as a result of no evidence for substantial DNA demethylation, 5hmC is considered as a stable epigenetic mark. Colquitt et al. found that TET3 oxidation product 5hmC elevates over gene bodies during mature olfactory sensory neurons (mOSNs) development and gene-body 5hmC patterning occurs between the progenitor and mOSNs stages. Additionally, this finding suggested that gene-body 5hmC patterning plays a physiologically significant role in the maintenance of stable cellular identities and transcriptional facilitation independent of its acting as an intermediate in DNA demethylation [64]. Zhang et al have revealed that TET1 possesses a specific function in the control of neural progenitor cells (NPCs) proliferation by promoting DNA demethylation and gene expression in adult brain. They observed that TET1-deficient mice displayed impaired hippocampal neurogenesis and cognitive deficiency such as memory and learning [65]. Xu et al. have uncovered that Xenopus Tet3 plays a vital role in early eye and neural development by two mechanisms. TET3′ enzymatic activity that modulates the 5mC/5hmC status at target gene promoter is an essential mechanism. The TET3 CXXC domain-mediated binding to unmethylated cytosines at target gene promoters provides another mechanism of regulating the transcription of TET3 target genes [66]. During oligodendrocyte (OL) development, three TET family proteins display unique subcellular and temporal expression patterns. 5hmC modification may act on the expression of critical genes required for OL maturation. Furthermore, siRNA-mediated silencing of TET1-3 proteins in OLs demonstrated that three TET proteins are necessary for OL differentiation [67]. As to the other oxidative derivatives of 5hmC, one study showed that 5fC and 5caC are transiently accumulated during differentiation of neural stem cells (NSCs) *in vitro* and *in vivo*. Moreover, enrichment of 5caC at the cell-type-specific promoters is observed during lineage specification of NSCs [68].

Taken together, these studies suggest that TET enzymes catalytic products, especially 5hmC, are likely multifunctional modification that is utilized either in the demethylation pathway or as a stable epigenetic mark itself, depending on the cell type and the genomic location of the modification during neural development (Table 1).

## THE NON-ENZYMATIC ACTIVITY OF TET FAMILY PROTEINS IN TUMORS AND NEURAL DEVELOPMENT

Epidemiological researches have demonstrated that chronic inflammation predisposes individuals to diverse types of tumor. Inflammation in the tumor microenvironment possesses versatile tumor-promoting effects. It is able to aid in the proliferation and survival of tumor cells, subvert adaptive immune responses, contribute to metastasis and angiogenesis, and alter responses to chemotherapeutic agents [69]. The hallmarks of cancer-related inflammation comprise many key inflammatory factors, such as IL-6. One study has shown that IL-6 level is highest in GBM cerebrospinal fluid (CSF) when compared with normal control low-grade glioma (LGG), and anaplastic astrocytomas (AA) [70]. Moreover, Kraus et al. have reported that 5hmC levels in gliomas are significantly lower compared to normal brain tissue and 5hmC values in GBM are lowest [23]. Intriguingly, Cao and his team have demonstrated that TET2 resolves inflammation *via* recruiting Hdac2 to selectively inhibit IL-6 in innate myeloid cells, which is independent of its enzymatic activity [12]. Since the inflammatory microenvironment contribute to the tumorigenesis and the development of tumors, we speculate that the inhibition of TET2 activities, including non-enzymatic activity and enzymatic activity of TET2 protein, may occur in tumors such as gliomas, and then the repression of TET2's non-enzymatic activity results in the elevation of IL-6 level in cancers.

In addition, the non-enzymatic role of TET proteins in transcription regulation has also been shown in other studies. Tsai et al. have found that TET1 interacts with hypoxiainducible factors (HIFs) to increase hypoxia-responsive gene expression and epithelial-mesenchymal transition (EMT) independent of its enzymatic activity [14]. TET2 boosts histone O-GlcNAcylation by directly interacting with OGT during gene transcription, which is independent of TET2 protein enzymatic activity [13]. The TET2 or TET3-OGT interaction promote GlcNAcylation and increase H3K4me3 through the SET1/COMPASS complex; OGT does not appear to influence TET proteins enzymatic activity [15]. TET1 interacts with the SIN3A co-repressor complex, leading to transcriptional repression of many TET1- targeted genes [16]. Moreover, Kaas et al. have showed that AAV-mediated overexpression of TET1 in the dorsal hippocampus partially impairs long-term memory formation independent of its enzymatic activity [71], which may be *via* the interaction between TET1 and some unknown proteins. Ambigapathy et al. showed that TET1 and ERK1/2 are critical partners during learning [72]. Rudenko et al. found that TET1 is critical for memory extinction [73]. However, in the two studies, there is little evidence indicating that we are able to make a distinction between catalytic activity and non- catalytic activity of TET1 in learning, but rather, that it is critical. Additionally, catalytically inactive TET3 mutant is able to partially rescue the effects on *Xenopus* eye and neural development after TET3 depletion, suggesting a non-enzymatic mechanism in gene regulation through recruiting or interacting with some unknown proteins [66]. Jin and his team have confirmed that Neuro2a cells have lower level of 5hmC; they have found that TET1 inhibits neuronal differentiation of Neuro2a cells independent of its catalytic activity *via* srGAP3, which is likely to be due to the interaction between TET1 and some unknown proteins [74].

**Figure 3:**
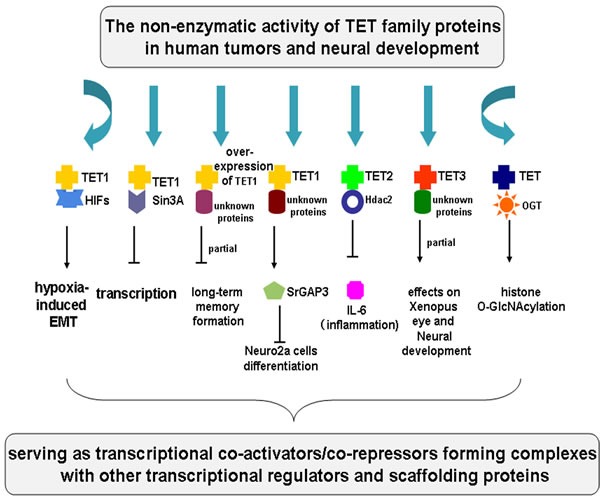
The non-enzymatic activity of TET family proteins in tumors and neural development The non-catalytic role of TET proteins in tumors and neural development is summarized. TET1 interacts with HIFs to increase hypoxia-responsive gene expression and EMT. TET1 interacts with the SIN3A co-repressor complex, leading to transcriptional repression of many TET1- targeted genes. Overexpression of TET1 impairs long-term memory formation and TET1 inhibits neuronal differentiation of Neuro2a *via* srGAP3, interacting with some unknown proteins. TET2 resolves inflammation *via* recruiting Hdac2 to selectively inhibit IL-6 in innate myeloid cells. TET3 is able to partially rescue the effects on *Xenopus* eye and neural development after TET3 depletion through recruiting or interacting with some unknown proteins. TET2 boosts histone O-GlcNAcylation by directly interacting with OGT during gene transcription. TET proteins might serve as transcriptional co-activators/co-repressors forming complexes with other transcriptional regulators and scaffolding proteins to perform important non-enzymatic functions in human tumors and neural development.

These studies suggest that TET proteins might serve as transcriptional co-activators/co-repressors forming complexes with other transcriptional regulators and scaffolding proteins to perform important non-enzymatic functions in human tumors and neural development (Figure 3).

## MECHANISMS AFFECTING BOTH ENZYMATIC ACTIVITY AND NON-ENZYMATIC ACTIVITY OF TET PROTEINS *VIA* INFLUENCING TET EXPRESSION IN TUMORS AND NEURAL DEVELOPMENT

A variety of mechanisms that interfere either directly or indirectly with TET expression, including TET mutations, upstream regulators of TET genes, methylation of TET genes, IDAX (also known as CXXC4), and multiple miRNAs, are able to have an effect on both enzymatic activity and non-enzymatic activity of TET family proteins in human tumors and neurodevelopment. In hematopoietic malignancies, including acute myeloid leukemias (AMLs), myeloproliferative neoplasms (MPN), myelodysplastic syndrome (MDS), and chronic myelomonocytic leukemia (CMML), TET2 gene has been shown to be frequently mutated [75–86]. The TET2 mutation frequencies in the different patient groups are MDS (6%-26%), CMML (20%-58%), primary and secondary AML (12%-32%), blastic plasmacytoid dendritic neoplasm (25%-54%), myeloproliferative neoplasms (MPNs) (2%-20%), B-cell (2%-12%) and T-cell (20%-83%) lymphomas, systemic mastocytosis (20-29%), and chronic myeloid leukemia (4%) [87]. However, mutations in TET genes present much less frequently in solid tumors such as clear-cell renal cell cancer, colorectal cancer and prostate cancer [88–91]. Sun et al. have reported that TET1 gene expression is dramatically induced upon depletion of high mobility group AT-hook2 (HMGA2) in invasive human breast cancer cells, suggesting that HMGA2 is an important upstream mediator of TET1 [92]. Furthermore, Huang and co-workers have found that TET1 expression is promoted *via* MLL-fusion proteins directly binding to the promoter region of TET1 in MLL-rearranged leukemia [93]. Two studies have reported that methylation of TET2 gene is infreguent in MPN and low-grade diffuse gliomas and may result in the inactivation of TET2 gene [94, 95]. Ko et al. have showed that IDAX expression lead to caspase activation and caspase-mediated cleavage could be involved in the degradation of TET2 by ubiquitylation [37]. Cheng et al. have reported that an extensive network of TET2-targeting miRNAs, such as miR-29b and miR-125a, could suppress TET2 expression and induce phenotypes related to hematopoietic malignancy [96]. Additionally, miR-22 mediates the repression of TET by a direct interaction between the TET genes and miR-22 in breast cancer [97]. MiR-494 has been reported to be able to inhibit TET1 gene expression through targeting its 3′UTR region in human hepatocellular carcinoma (HCC) [98]. Interestingly, miR-15b directly reduces TET3 expression to promote neurogenesis and repress neural progenitor proliferation during early neocortical development [99].

In addition to the above findings, TET proteins expression is elevated in proneural glioblastomas, leiomyoma, and diffuse intrinsic pontine glioma (DIPG), but the mechanisms that lead to the elevation of TET proteins are elusive in these studies [24, 25, 100]. In neuroblastoma cells, hypoxia lead to TET1 upregulation, and TET1 is required for full induction of hypoxia-responsive genes and global 5hmC level increases [101].

## CLINICAL IMPLICATIONS OF TET PROTEINS DUAL ACTIVITIES IN HUMAN TUMORS AND NEURAL DEVELOPMENT

Serials of investigations have discovered that TET proteins dual activities (catalytic or non-catalytic) exert tumor-suppressive or tumor-promoting functions in different types of tumors (Table 2). There are studies where TET proteins dual activities have or may have tumor-suppressing functions. Rasmussen and colleagues have proposed that TET2 catalytic activity prevents leukemic transformation and TET2 mutations promote leukemogenesis [102]. In prostate cancer, compared to patients with high TET1 mRNA levels, patients with low TET1 mRNA levels possess a doubled risk at developing metastases; TET1 catalytic activity plays a suppressive role in prostate cancer [91]. In breast cancer, TET1 suppresses tumor growth, intravasation, and metastasis dependent of its catalytic activity [92]. Moreover, TET1 inhibits tumor development and invasion partly *via* maintaining the expression of tissue inhibitors of metalloproteinase (TIMP) dependent of its catalytic activity [103]. In addition, TET2 may suppress the tumor-promoting effects of inflammation through recruiting Hda2 to inhibit IL-6 independent of its catalytic activity [12]. Furthermore, TET1 could suppress gastric cancer formation *via* combining DNA demethylation with inhibition of oncogenic protein EZH2 and DNA-PK activation of p53 [104]. However, there are studies where TET proteins dual activities have or may have tumor-promoting functions. TET1 catalytic activity plays an indispensable oncogenic role through coordination with MLL-fusion proteins in MLL-rearranged leukemia [93]. In proneural glioblastomas, TET1-catalytic production of 5hmC is required for increased tumorigenicity [25]. In DIPG, TET proteins (TET1 and TET3) and 5hmC may underlie the tumor formation and resistance to treatment [24]. Furthermore, TET1 and TET3 protein levels are higher in uterine leiomyoma, and TET1 or TET3 knockdown decrease cell proliferation of leiomyoma cells [100]. Interestingly, TET1 interacts with HIF-1α and HIF-2α to enhance epithelial-mesenchymal transition (EMT) independent of its enzymatic activity and then could contribute to cancer metastasis [14]. Christopher et al. have observed that, in hypoxia, TET1 expression and 5hmC level increase within tumorigenic, N-type neuroblastoma cells [101]. These findings showed that TET proteins dual activities (catalytic or non-catalytic) are capable of playing dual roles (tumor-suppressing or tumor-promoting) *via* interacting with the TET binding protein partners in different types of tumors. Therefore, TET proteins can be a promising molecular target for tumors therapy. When TET proteins serve as the tumor suppressors, we contribute to the expression of them. When TET proteins serve as the tumors promoters, we suppress the expression of them.

**Table 2:** Clinical implications of TET proteins dual roles in tumors

Tumors	5hmC levels	TET proteins	Role in tumorigenesis	Ref
many hematopoietic malignancies	↓	(TET2)↓	tumor suppression	[75–87,102]
breast cancer	↓	(TET1)↓	tumor suppression	[92]
prostate cancer	↓	(TET1)↓	tumor suppression	[91]
human hepatocellular carcinoma	↓	(TET1)↓	tumor suppression	[98]
gastric cancer	↓	(TET1)↓	tumor suppression	[104]
MLL-rearranged leukemia	↑	(TET1)↑	tumor promotion	[93]
diffuse intrinsic pontine glioma	↑	(TET1 and TET3)↑	tumor promotion (not determined)	[24]
proneural glioblastomas	↑	(TET1)↑	tumor promotion	[25]
uterine leiomyoma	↑	(TET1 and TET3)↑	tumor promotion	[100]
neuroblastoma in hypoxia	↑	(TET1)↑	tumor promotion	[101]
inflammation-induced tumors	—	(TET2)—	tumor suppression (not determined)	[12]

Note: ↑: higher levels compared to normal tissue; ↓: lower levels compared to normal tissue; —: possible no alteration compared to normal tissue

In neural development, TET proteins dual activities (catalytic or non-catalytic) also exert dual functions (promoting or suppressing). TET proteins catalytic activity plays a promoting role in neuronal differentiation, mOSN development, adult neural progenitor cell proliferation, Xenopus eye and neural development, and OL development [27, 64–67]. In addition, TET3 non-catalytic activity plays a promoting role in Xenopus eye and neural development [66]. However, TET1 non-catalytic activity plays a suppressing role in neuronal differentiation of Neuro2a cells [74]. The elucidation of the relationship between the TET proteins dual activities and dual roles in neural development might offer potential advances in treatments for brain disorders.

## CONCLUSIONS AND OUTLOOKS

As summarized in this review, extensive researches on TET family proteins have shed light on their enzymatic activity and non-enzymatic activity in neural development and human tumors. Further understanding of TET enzymes dual activities in normal neurodevelopment could make us realize that defects or even subtle aberrations of TET proteins in neurological disorders, such as human tumors. TET proteins dual activities are capable of playing dual roles in human tumors, including tumor-suppressive and tumor-enhancing effects. Some of presented findings indicate that the great potential and relevance of TET family proteins as the novel classes of therapeutic target in human tumors.

## Abbreviations

TET: ten-eleven translocation
5mC: 5-methylcytosine
5hmC: 5-hydroxymethylc-ytosine
5fC: 5-formylcytosine
5caC: 5-carboxylcytosine
TDG: thymine DNA g-lycosylase
BER: base-excision repair
MLL: mixed lineage leukemia
AML: acute myeloid leukemia
5hmU: 5-hydroxymethyluridine
AID: activation-induced deaminase
APOBEC: apolioprotein B mRNA-editing enzyme catalytic polypeptide
Hdac: histone deacetylase
OGT: O-glcNAC- transferase
HIFs: hypoxiainducible factors
IDH: isocitrate dehydrogenase
DIPG: diffuse intrinsic pontine glioma
GBM: glioblastoma multiforme
NPCs: neural precursor cells
OL: oligodendrocyte
NSCs: neural stem cells
DSBH: double-stranded β-helix
α-KG: α-ketoglutarate
DNMTs: DNA methyltransterases
2-HG: 2-hydroxyglutarate
mOSNs: mature olfactory sensory neurons
CSF: cerebrospinal fluid
LGG: low-grade glioma
AA: anaplastic astrocytomas
MPN: myeloproliferative neoplasms
MDS: myelodysplastic syndrome
CMML: chronic myelomonocytic leukemia
HMGA2: high mobility group AT-hook2
HCC: hepatocellular carcinoma
TIMP: tissue inhibitors of metalloproteinase
EMT: epithelial-mesenchymal transition
